# ESC Joint Working Groups on Cardiovascular Surgery and the Cellular Biology of the Heart Position Paper: Peri-operative myocardial injury and infarction in patients undergoing coronary artery bypass graft surgery

**DOI:** 10.1093/eurheartj/ehx383

**Published:** 2017-07-25

**Authors:** Matthias Thielmann, Vikram Sharma, Nawwar Al-Attar, Heerajnarain Bulluck, Gianluigi Bisleri, Jeroen JH Bunge, Martin Czerny, Péter Ferdinandy, Ulrich H. Frey, Gerd Heusch, Johannes Holfeld, Petra Kleinbongard, Gudrun Kunst, Irene Lang, Salvatore Lentini, Rosalinda Madonna, Patrick Meybohm, Claudio Muneretto, Jean-Francois Obadia, Cinzia Perrino, Fabrice Prunier, Joost P.G. Sluijter, Linda W. Van Laake, Miguel Sousa-Uva, Derek J. Hausenloy

**Affiliations:** 1 Department of Thoracic and Cardiovascular Surgery, West-German Heart and Vascular Center, University Hospital Essen, Hufelandstraße 55, 45122, Essen, Germany; 2 Department of Internal Medicine, Cleveland Clinic, 9500 Euclid Avenue, Cleveland, Ohio 44195, USA; 3 The Hatter Cardiovascular Institute, University College London, 67 Chenies Mews, London WC1E 6HX, UK; 4 Scottish National Advanced Heart Failure Service, Golden Jubilee National Hospital, Agamemnon Street, G81 4DY, Clydebank, UK; 3 The Hatter Cardiovascular Institute, University College London, 67 Chenies Mews, London WC1E 6HX, UK; 5 Division of Cardiac Surgery, Queen’s University, 99 University Avenue, Kingston, Ontario K7L 3N6, Canada; 6 Department of Intensive Care, Erasmus Medical Center,'s-Gravendijkwal 230, 3015 CE Rotterdam, Holland; 7 Department of Cardiac Surgery, University Heart Center Freiburg-Bad Krozingen, Hugstetterstrasse 55, Freiburg, D-79106, Germany; 8 Department of Pharmacology and Pharmacotherapy, Semmelweis University, Üllői út 26, H - 1085 Budapest, Hungary; 9 Pharmahungary Group, Szeged, Graphisoft Park, 7 Záhony street, Budapest, H-1031, Hungary; 10 Department of Anaesthesia and Intensive Care Medicine, University Hospital Essen, Hufelandstr. 55, 45122 Essen, Germany; 11 Institute for Pathophysiology, West German Heart and Vascular Center, University of Essen Medical School, Hufelandstr. 55, 45122 Essen, Germany; 12 University Clinic of Cardiac Surgery, Innsbruck Medical University, Christoph-Probst-Platz 1, Innrain 52, A-6020 Innsbruck, Austria; 11 Institute for Pathophysiology, West German Heart and Vascular Center, University of Essen Medical School, Hufelandstr. 55, 45122 Essen, Germany; 13 Department of Anaesthetics, King’s College Hospital and King’s College London, Denmark Hill, London, SE5 9RS, UK; 14 Internal Medicine II, Division of Cardiology, Medical University of Vienna, Währinger Gürtel 18-20, 1090, Vienna, Vienna, Austria; 15 Department of Cardiac Surgery, The Salam Center for Cardiac Surgery, Soba Hilla, Khartoum, Sudan, Italy; 16 Center of Aging Sciences and Translational Medicine—CESI-Met and Institute of Cardiology, Department of Neurosciences, Imaging and Clinical Sciences “G. D”'Annunzio University, Via dei Vestini, 66100 Chieti, Italy; 17 The Center for Cardiovascular Biology and Atherosclerosis Research, Department of Internal Medicine, The University of Texas Medical School at Houston, 6431 Fannin Street, MSB 1.240, Houston, TX 77030, USA; 18 Department of Anaesthesiology, Intensive Care Medicine and Pain Therapy, University Hospital Frankfurt, Theodor-Stern-Kai 7, 60590 Frankfurt am Main, Germany; 19 Department of Cardiac Surgery, University of Brescia Medical School. P.le Spedali Civili, 1., Brescia, 25123, Italy; 20 Department of Cardiothoracic Surgery, Louis Pradel Hospital, 28 Avenue du Doyen Jean Lépine, 69677 Bron Cedex, Lyon, France; 21 Division of Cardiology, Department of Advanced Biomedical Sciences, Federico II University, Corso Umberto I 40 - 80138 Naples, Italy; 22 Department of Cardiology, Institut MITOVASC, University of Angers, University Hospital of Angers, 2 rue Lakanal, 49045 Angers Cedex 01, Angers, France; 23 Cardiology and UMC Utrecht Regenerative Medicine Center, University Medical Center Utrecht, Heidelberglaan 100, 3584CX, Utrecht, The Netherlands; 24 Department of Cardiology, Division of Heart and Lungs and Regenerative Medicine Center, University Medical Center Utrecht, Heidelberglaan 100, 3584 CX Utrecht, The Netherlands; 25 Department of Cardiothoracic Surgery, Hospital da Cruz Vermelha, Lisbon, Portugal; 3 The Hatter Cardiovascular Institute, University College London, 67 Chenies Mews, London WC1E 6HX, UK; 26 The National Institute of Health Research University College London Hospitals Biomedical Research Centre, Maple House Suite A 1st floor, 149 Tottenham Court Road, London W1T 7DN, UK; 27 Cardiovascular and Metabolic Disorder Research Program, Cardiovascular and Metabolic Disorders Program, Duke-National University of Singapore, 8 College Road, Singapore 169857, Singapore; 28 National Heart Research Institute Singapore, National Heart Centre Singapore, 5 Hospital Drive, Singapore 169609, Singapore; 29 Yong Loo Lin School of Medicine, National University Singapore, 1E Kent Ridge Road, Singapore 119228, Singapore; 30 Barts Heart Centre, St Bartholomew's Hospital, West Smithfield, London, EC1A 7BE, UK

## Introduction

Coronary artery disease (CAD) is one of the leading causes of death and disability in Europe and worldwide. For patients with multi-vessel CAD, coronary artery bypass graft (CABG) surgery is a common approach for coronary revascularization, and is of proven symptomatic and prognostic benefit. Due to an aging population, higher prevalence of co-morbidities (such as diabetes mellitus, heart failure, hypertension, and renal failure), and a growing requirement for concomitant surgical procedures (such as valve and aortic surgery), higher risk patients are undergoing surgery.^1–3^ This has resulted in an increased risk of peri-operative myocardial injury (PMI)^4^ and Type 5 myocardial infarction (MI), both of which are associated with worsened clinical outcomes following CABG surgery. The aetiology and determinants of PMI and Type 5 MI are multi-factorial (see *Tables 1 *and* 2* for summary). Although diagnostic criteria have been proposed for Type 5 MI (based on an elevation in cardiac biomarkers in the 48-h post-operative period and electrocardiogram/angiography/imaging evidence of MI^5^^,^^13^), there is currently no clear definition for prognostically significant PMI, in terms of the level of post-operative cardiac biomarker elevation, which is associated with worsened clinical outcomes following CABG surgery.

**Table 1:** Causes of peri-operative myocardial injury in patients undergoing coronary artery bypass graft surgery

**Injury related to primary myocardial ischaemia (mainly graft-related)** Plaque rupture in native coronary artery or graft Thrombus formation in the native coronary artery or graft Acute graft failure due to occlusion, kinking, overstretching, anastomotic stenosis or spasm of the grafted blood vessel Arterial graft spasm **Myocardial injury related to unfavourable haemodynamics or oxygen supply** Tachyarrhythmia Cardiogenic or hypovolaemic shock Severe respiratory failure Severe anaemia Left ventricular hypertrophy Coronary artery or graft micro-embolism Inadequate cardioprotection from cardioplegia **Myocardial injury not related to myocardial ischaemia** Cardiac handling during surgery Direct injury to the myocardium Surgical myectomy Inflammatory injury due to cardiopulmonary bypass **Multifactorial or indeterminate myocardial injury** Heart failure Severe pulmonary embolism Sepsis Critically ill patients Renal failure

Adapted from reference 6.

**Table 2:** Predictors of peri-operative myocardial infarction/graft-failure

**Patient factors** Advanced age^6^ Female sex^7^ Impaired LV systolic function prior to surgery^6^ Left main stem or 3-vessel CAD^6^^,^^7^ Pre-operative MI^6^ Unstable angina^6^^,^^8^^,^^9^ Previous history of coronary revascularisation Poor target coronary artery quality^6^^,^^10^ Uncontrolled hyperglycaemia^10^^,^^11^ EUROSCORE >6^9^ **Surgery factors** Longer surgery time^6^ Prolonged cardio-pulmonary bypass and/or aortic cross clamp time^6^^,^^8^^,^^9^^,^^11^ Coronary endarterectomy Concomitant aortic and/or valve surgery Inadequate myocardial protection during CABG^12^ Incomplete revascularisation^9^ Poor vein graft quality Small internal thoracic artery

Therefore, the aim of this European Society of Cardiology (ESC) Joint Working Groups (WG) Position Paper is to provide a set of recommendations to better define the level of cardiac biomarker elevation following CABG surgery at which PMI should be considered prognostically significant, and therefore prompt further clinical evaluation. We also provide guidance on how to manage patients with PMI and Type 5 MI.

## Defining type 5 myocardial infarction

Type 5 MI has been defined in the Third Universal Definition of MI (2012) as an elevation of cardiac troponin (cTn) values >10× 99th percentile upper reference limit (URL) during the first 48 h following CABG surgery, in patients with normal baseline cardiac cTn values (<99th percentile URL) together with either: (a) new pathological Q waves or new left bundle branch block (LBBB), or (b) angiographic documented new graft or new native coronary artery occlusion, or (c) imaging evidence of new loss of viable myocardium or new regional wall motion abnormality (RWMA).^13^ In general, Type 5 MI is mainly due to an ischaemic event arising from either a failure in graft function, an acute coronary event involving the native coronary arteries, or inadequate cardioprotection. The incidence of Type 5 MI following CABG surgery varies depending on the diagnostic criteria which are used to define it. When assessed by elevations in cardiac biomarkers and new electrocardiogram (ECG) evidence of Q waves or LBBB, the incidence has been reported to range from 5 to 14%,^4^ whereas it ranges from 20 to 30% when using cardiac magnetic resonance (CMR) to detect new loss of viable myocardium.^14–16^

The current definition of Type 5 MI does have several limitations:

1. The selection of a cTn elevation of 10× URL as a threshold for diagnosing Type 5 MI was arbitrarily chosen. Elevated cTn of 10× URL occurs in over 90% of all patients undergoing CABG surgery.^8^^,^^12^
2. Type 5 MI requires the presence of ECG/angiography/imaging evidence of MI, and ignores post-surgical isolated elevations in cardiac biomarkers which may still be prognostically significant (i.e. biomarker elevations in the absence of ECG/angiographic or other imaging evidence of MI).
3. The diagnostic criteria for Type 5 MI can also be quite challenging in the setting of CABG surgery for several reasons: (i) In a substantial number of patients, the ECG may not be interpretable and many of the ECG changes following CABG surgery may be non-specific for MI.^15–17^ (ii) Coronary angiography is rarely performed post-surgery to diagnose very early graft failure; and (iii) Echocardiography is the most practical imaging modality for detecting new loss of viable myocardium or new RWMA following CABG surgery, but it may not be diagnostic in many cases.

As such, the diagnosis of Type 5 MI in the 48 h post-operative period may be quite challenging, unless it presents with obvious graft failure or a significant ischaemic event. Therefore, in many cases, patients may sustain prognostically significant PMI, but this may be overlooked. The Society for Cardiovascular Angiography and Interventions (SCAI) has proposed a new definition for clinically relevant MI, which takes into account isolated elevations in either creatine kinase-MB fraction (CK-MB) or cTn within 48 h of CABG surgery.^18^ With respect to CK-MB, these recommendations propose a peak elevation ≥10× URL in isolation or ≥5× URL with new pathologic Q-waves in ≥2 contiguous ECG leads or new persistent LBBB. A substantially higher cut-off for cTn elevation of ≥70× URL in isolation or ≥35× URL with new pathologic Q-waves in ≥2 contiguous ECG leads or new persistent LBBB is also proposed in that paper.^18^ Again, these threshold levels were arbitrarily chosen, and further studies are required to validate their new definition of clinically relevant MI, and explore their relationship to clinical outcomes post-surgery. In addition, these recommendations do not take into consideration isolated elevations of cardiac biomarkers below these thresholds, which may still be clinically relevant and prognostically significant.

## Defining peri-operative myocardial injury

Peri-operative myocardial injury is defined as an isolated elevation in cardiac biomarkers (CK-MB and/or cTn) greater than the upper limit of normal, in the 48-h post-operative period. However, this level of cardiac biomarker elevation occurs in virtually all patients undergoing CABG surgery, and there is no clear consensus on the level of cardiac biomarker elevation above which, it is either clinically relevant or prognostically significant. A recent publication has proposed defining PMI as an isolated elevation in cTn <10× the URL within 48 h of CABG surgery,^5^ but this definition does not include those patients who have isolated cTn elevations >10× URL in the absence of ECG/angiographic or other imaging evidence of MI. Therefore, in this ESC Joint WG Position Paper we provide recommendations for defining prognostically significant PMI following CABG surgery, which should prompt further clinical evaluation to exclude Type 5 MI. In this paper, we mainly focus on those patients undergoing elective isolated on-pump or off-pump CABG surgery, as the presence of prognostically significant PMI is more challenging to define in patients presenting with an acute coronary syndrome (with elevated pre-operative cardiac biomarkers), and those having concomitant valve or aortic surgery. However, patients presenting with an acute coronary syndrome are become increasingly rare since many undergo primarily percutaneous intervention.

### Isolated elevations in creatine kinase-MB fraction and mortality post-coronary artery bypass graft surgery

**Figure 1:**
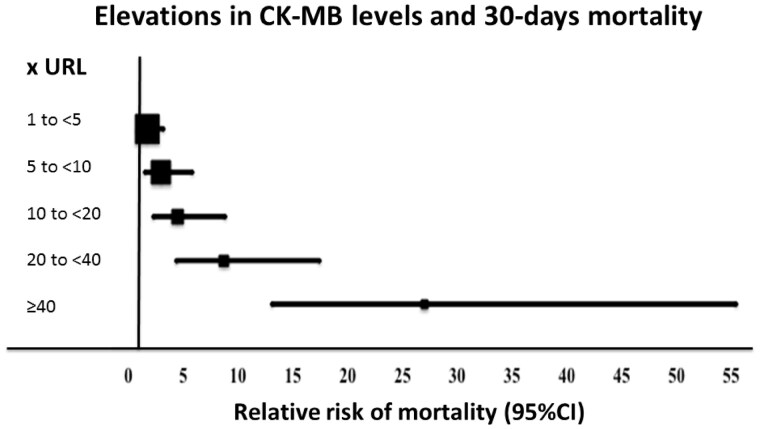
Relationship between creatine kinase-MB fraction elevation post**-**coronary artery bypass graft surgery with relative risk of mortality at 30 days (adapted from meta-analysis by Domanski *et al.*^29^).

A large number of early studies have assessed the prognostic significance of isolated elevations in CK-MB following CABG surgery in the absence of ECG/angiographic or other imaging evidence of MI (*Table 3* and *Figure 1*). These studies have demonstrated a graded increase in short, medium, and long-term mortality beginning with an isolated CK-MB elevation ≥3× URL within 24 h of CABG surgery. Above isolated 10× URL elevations, there appears to be a progressive increase in short-term (30 days) and longer-term mortality (1 year and over), which is independent of other evidence of MI.^20^^,^^23^^,^^29^ In most centres, CK-MB has now been replaced by the use of cardiac troponins, as the latter are more sensitive and specific for detecting PMI and Type 5 MI following CABG surgery.^32^^,^^33^ Hence, we have elected to not use isolated CK-MB elevations post-surgery to define prognostically significant PMI.

**Table 3:** Major recent studies showing elevations in creatine kinase-MB fraction to be associated with mortality post-coronary artery bypass grafting surgery

Study	Type of study and surgery	Number of patients	Cardiac biomarker (time)	Time from CABG when biomarker level taken	Major findings
Costa *et al.*^19^ (ARTS trial)	Multi-centre prospective study CABG only	496	CK-MB	6,12,18 h	<1× URL 0.0% 30 d mortality 1.1% 1 yr mortality 1–3× URL 0.5% 30 d mortality 0.5% 1 yr mortality ≥3–5× URL 5.4% 30 d mortality 5.4% 1 yr mortality >5× URL 7.0% 30 d mortality 10.5% 1 yr mortality
Klatte *et al.*^20^ (GUARDIAN Trial)	Multi-centre prospective study CABG only	2394	CK-MB ECG	8, 12, 16, 24 h	<5× URL 3.4% 6 mth mortality (RR 1.0) ≥5–10× URL 5.8% 6 mth mortality (RR 1.69) ≥10–20× URL 7.8% 6 mth mortality (RR 2.28) ≥20× URL 20.2% 6 mth mortality (RR 5.94 >5× URL + new Q waves worse 6 mth mortality (8.0% vs. 3.1%)
Steuer *et al.*^21^	Prospective single centre, CABG only	4911	CK-MB	24 h	>61 ug/L Relative Hazard 1.3 to 1.4 for late mortality (up to 6 years)
Brener *et al.*^12^	Retrospective single centre analysis, CABG only	3812	CK-MB	24 h	≤1× URL 7.2% 3 yr mortality 1–3× URL 7.7% 3 yr mortality 3–5× URL 6.3% 3 yr mortality 5–10× URL 7.5% 3 yr mortality >10× URL 20.8% 3 yr mortality >10× URL predicted 3 yr mortality (HR 1.3)
Marso *et al.*^22^	Single centre registry post-hoc analysis CABG only	3667	CK-MB	Single measurement mean 15.2 h	≤1× URL 0.6% 30 d mortality >1–3× URL 1.1% 30 d mortality >3× URL 2.2% 30 d mortality >4× URL associated with increased long-term mortality 5.1 yr (RR 1.3)
Ramsay *et al.*^23^	Multi-centre prospective randomized trial CABG only	800	CK-MB	4,8, 16, 20,24, 30, 36 h Day 2, 4, 7, 30	0–5× URL 0.9% 30 d mortality 5–10× URL 0.7% 30 d mortality 10–20× URL 0.9% 30 d mortality >20× URL 6.0% 30 d mortality AUC and peak CK-MB correlated very well.
Engoren *et al.*^24^	Retrospective analysis CABG only	1161	CK-MB	10–18 h	>8× URL HR 1.3 increased 1 yr mortality
Newall *et al.*^7^	Observational cohort study CABG only	2860	CK-MB	Single value up to 24 h	3–6× URL HR 2.1 for 1 yr mortality >6× URL HR 5.0 for 1 yr mortality
Mahaffey *et al.*^25^	Pooled analysis of four trials CABG only	1406	CK-MB	Single value up to 24 h	<3× URL 2.5% 30 d mortality; 3.7% 6 mth mortality 3–5× URL 2.9% 30 d mortality; 4.7% 6 mth mortality 5–8× URL 3.1% 30 d mortality; 6.1% 6 mth mortality ≥8× URL 8.6% 30 d mortality; 9.6% 6 mth mortality
Muehlschlegel *et al.*^26^	Prospective single centre study CABG only	545	CK-MB	Daily from day 1 to 5	24 h 1.23 for each 25 mg/L increase of 5 yr mortality ECG changes alone did not predict 5 year mortality.
Petaja *et al.*^27^	Meta-analysis CABG and/or valve surgery	21 657	CK-MB	Variable (peak or absolute value at various time points post-op)	CK-MB ≥5× URL –RR of short term mortality 3.69% (CI 2.17–6.26); RR of long term (6–60 m) mortality 2.66% (CI 1.95–3.63)
Vikenes *et al.*^28^	Prospective single centre study CABG and/or valve surgery	205	CK-MB	1–3, 4–8, 24, 48 and 72 h	CK-MB elevation ≥ 5× URL was associated with worst long term event free survival (median follow-up 92 mths).
Domanski *et al.*^29^	Meta-analysis CABG only	18 908	CK-MB (<24 h)	Single value < 24 h	1–5× URL 1.69% RR of 30 d mortality 5–10× URL 2.98% RR of 30 d mortality 10–20× URL 4.47% RR of 30 d mortality 20–40× URL 8.73% RR of 30 d mortality ≥40× URL 27.01% RR of 30 d mortality CK-MB levels were significantly associated with 1 year mortality; there was a non-significant trend for association with 5 year mortality
Søraas *et al.*^30^	Registry analysis, single centre study CABG only	1350	CK-MB cTnI	7,20, 44 h	There was no difference in mortality between those with CK-MB ≥7.8× URL vs. ≤4× URL CK-MB levels at 44 h postoperatively had a greater predictive value for mortality than at 7 or 20 h. Peak CK-MB levels predicted long-term mortality (median 6.1 years) after univariate but not multivariate analysis (including cTnI).
Farooq *et al.*^31^ SYNTAX trial substudy	Post hoc analysis of SYNTAX trial data; CABG only	474	CK-MB	6, 12 h (CK-MB was measured only if CK ≥ 2× URL	CK-MB <3/≥3× URL separated patients into low and high-risk groups based on 4-year mortality (All-cause mortality 2.3% vs. 9.5% *P* = 0.03). CK-MB ≥3× URL was associated with significantly higher frequency of high SYNTAX Score tertile (≥33)

AUC, area under the curve; CABG, coronary artery bypass grafting; CMR, cardiac MRI; CK-MB, creatine kinase-MB fraction; d, day; ECG, electrocardiogram; ECHO, echocardiocardiogram; HR, hazards ratio; h, hour; LGE, late gadolinium enhancement; LV, left ventricle; MACE, major adverse cardiac events; MI, myocardial infarction; mth, month; ng, nanogram; ONBEAT, on-pump beating heart; CABG ONSTOP, on-pump CABG; OR, odds ratio; post-op, post-operative; PMI, perioperative myocardial injury; RR, relative risk; TEE, transoesophageal echocardiogram; cTnI, Troponin I; cTnT, Troponin T; UA, unstable angina; URL, upper reference limit; yr, year.

### Isolated elevations in cTnT and cTnI and mortality post-coronary artery bypass graft surgery

Cardiac troponins have greater sensitivity and specificity for myocardial necrosis, when compared to CK-MB, and have been found to be superior to CK-MB in predicting mortality post-CABG surgery.^30^^,^^34–37^ However, the interpretation of isolated changes in cTn levels in the post-operative period, in the absence of ECG/angiographic or other imaging evidence of MI, can be quite challenging given the different cTn assays used, the introduction of high-sensitive assays for cTn, and the presence of renal dysfunction.

**Figure 2:**
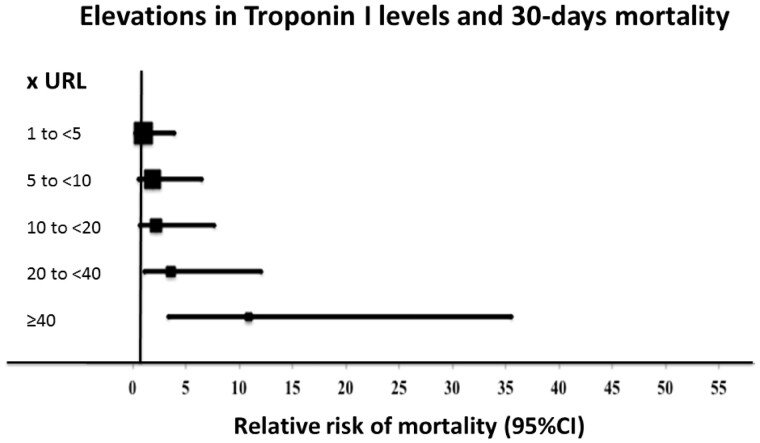
Relationship between Troponin I elevation post-coronary artery bypass graft surgery with relative risk of mortality at 30 days (adapted from meta-analysis by Domanski *et al.*^29^).

As with CK-MB, there appears to be a graded increase in short-term and long-term mortality following CABG surgery, based on the magnitude of post-operative cTnI or cTnT levels (*Tables 4 *and* 5*). Overall, there is a clear association between isolated elevations of cTnT ≥7× URL^41^ and cTnI levels ≥20× URL^29^^,^^41^ with significant increases in short-term (30 days) and long-term (one year and over) mortality after CABG surgery (*Tables 4, 5* and *Figure 2*). Importantly, these findings were shown to be independent of ECG/angiography/imaging evidence of MI, confirming that isolated elevations of cTn following CABG surgery can predict mortality. The studies that have been used to define these thresholds used various generations of ‘standard’ cTnT and cTnI assays, and currently there is lack of sufficient data to accurately determine these thresholds for the high sensitivity-cTnT or cTnI assays. Hence, the above threshold for cTnT does not apply to the high-sensitive cTnT assay, and so for this assay, additional ECG and/or imaging evidence of MI appears to be required to identify those CABG patients at a higher risk of mortality when ≥10× URL hs-cTnT elevation is measured.^8^ The majority of studies have reported isolated elevations between 24 and 48 h post-surgery as being the most discriminatory for predicting clinical outcomes.^27^^,^^30^^,^^36–38^^,^^42^ Whether it is necessary to measure the AUC cTn elevation or whether a single time-point measurement of cTn is sufficient to predict post-surgical outcomes, is not clear. Recent evidence suggests that the AUC of high-sensitive cTnT may be a good surrogate for MI size.^54^

**Table 4:** Major recent studies showing elevations in Troponin T to be associated with mortality post-coronary artery bypass grafting surgery

Study	Type of study and surgery	Number of patients	Cardiac biomarker (time)	Time from CABG when biomarker level taken	Major findings
Januzzi *et al.*^36^	Prospective single centre study CABG only	224	cTnT CK-MB	Immediately post-op, 6–8 h and 18–24 h	cTnT level in the highest quintile (≥1.58 ng/mL; ≥15× URL) immediately post-op or at 18–24 h predicted in-hospital death. CK-MB levels did not offer additional prognostic benefit to cTnT in multivariate analysis
Lehrke *et al.*^38^	Prospective single centre study CABG and/or valve surgery	204	cTnT	4, 8 h then every day for 7 days	cTnT >0.46 μg/L (>46× URL) at 48 h after surgery was the optimum discriminator for long-term cardiac mortality (28 mths, OR 4.93)
Kathiresan *et al.*^37^	Prospective single centre study CABG only	136	cTnT CK-MB	Immediately post-op, 6–8 h and 18–24 h post-op	cTnT >1.58 μg/L at 18–24 h was the optimum discriminator for 1 year cardiac mortality (OR 5.45) Elevations in CK-MB were not predictive of mortality
Nesher *et al.*^39^	Retrospective observational single centre study Cardiac surgery (CABG and/or valve)	1918	cTnT	Single sample <24 h	cTnT level ≥0.8 μg/L (8× URL) was most discriminatory for MACE (30 day death, electrocardiogram-defined infarction, and low output syndrome) (OR 2.7) 0–3.9× URL 0.5% 30 day mortality 5–5.9× URL 1.6% 30 day mortality 6–7.9× URL 1.0% 30 day mortality 8–12.9× URL 1.8% 30 day mortality >13× URL 6.8% 30 day mortality
Muehlschlegel *et al.*^26^	Retrospective analysis CABG only	1013	cTnT	Daily from day 1 to 5	24 h cTnT rise > 110× URL HR 7.2 of 5 yr mortality cTnT at 24 h were independent predictors of 5 year mortality in a multivariate model (No additional benefit of measuring cTn beyond 24 h). Majority of patients had peak cTnI and CK-MB levels at 24 h. ECG changes alone did not predict 5 year mortality.
Mohammed *et al.*^40^	Prospective single centre study, retrospective analysis CABG only	847	cTnT	6–8 and 18–24 h	A cTnT of < 1.60 (<160× URL) had good negative predictive value for poor 30 day outcomes (death or heart failure)
Petaja *et al.*^41^	Meta-analysis CABG and/or valve surgery	2,547	cTnT	<48 h post op	≥7–16× URL: Short term mortality 3.2% vs. 0.5% for <7–16× URL elevation (RR 4.68–6.4); Long term mortality (12–28 mth) 16.1% vs. 2.3% (RR 5.7–10.09). (Pooled RR of mortality could not be calculated)
Søraas *et al.*^30^	Registry analysis, single centre study CABG only	1,350	cTnT CK-MB	7,20, 44 h post op	Patients with peak cTnT ≥ 5.4× URL had much higher long-term mortality (median 6.1 years) than those with <5.4× URL cTnT elevation. cTnT levels at 44 h postoperatively had a greater predictive value for long-term mortality than at 7 or 20 h. Peak Trop T levels predicted long-term mortality after multivariate analysis.
Wang *et al.*^8^	Retrospective analysis CABG only	560	hs-cTnT ECG/ECHO changes	12–24 h after CABG	In a multivariate model >10× URL rise in hs-TNT + ECG/ECHO evidence of recent MI or regional ischaemia predicted 30 day (HR 4.9) and long-term mortality (median follow-up 1.8 years) (HR 3.4). > 10× URL rise in hs-cTnT was seen in 90% patients.
Gober *et al.*^42^	Retrospective study from registry data CABG only	290	cTnT CK-MB	8,16 h post op	cTnT > 0.8 ng/mL (>80× URL) at 6–8 h was predictive of in hospital adverse outcomes and long term (4yr) mortality (OR 4.0). However, cTnT measured at 6–8 h was inferior to cTnT taken at 20 h in its prognostic ability.

AUC, area under the curve; CABG, coronary artery bypass grafting; CMR, cardiac MRI; CK-MB, creatine kinase-MB fraction; d, day; ECG, electrocardiogram; ECHO, echocardiocardiogram; HR, hazards ratio; h, hour; LGE, late gadolinium enhancement; LV, left ventricle; MACE, major adverse cardiac events; MI, myocardial infarction; mth, month; ng, nanogram; ONBEAT, on-pump beating heart; CABG ONSTOP, on-pump CABG; OR, odds ratio; post-op, post-operative; PMI, perioperative myocardial injury; RR, relative risk; TEE, transoesophageal echocardiogram; cTnI, Troponin I; cTnT, Troponin T; UA, unstable angina; URL, upper reference limit; yr, year.

**Table 5:** Major recent studies showing elevations in Troponin I to be associated with mortality post-coronary artery bypass grafting surgery

Study	Type of study and surgery	Number of patients	Cardiac biomarker (time)	Other features	Major findings
Greenson *et al.*^43^	Single centre prospective study; CABG or Aortic valve replacement	100	cTnI CK-MB	Pre-op, 24 h and 48 h, then daily until discharge or 1 week	Peak cTnI > 60 ng/mL (> 120× URL) predictive of cardiac events up to 30 days post op
Holmvang *et al.*^35^	Single centre prospective study, CABG only	103	cTnT cTnI CK-MB Myoglobin	Every 2 h in first 20 h, 24, 30, 36 and 48 h, 72 and 98 h	ECG changes unable to differentiate between patients with or without graft failure. CK-MB and cTnT (but not cTnI or Myoglobin) levels were significantly higher in patients with graft failure vs. those without. Optimal discrimination values were 30 mcg/L for CK-MB (sensitivity 67%, specificity 65%) and 3 mcg/L for cTnT (sensitivity 67%, specificity 76%). In multivariate analysis cTnT > 3 mcg/L was significantly associated with graft failure (sensitivity of 75% compared to 20% for clinical criteria)
Eigel *et al.*^44^	Prospective single centre study; CABG only (Excluded MI within 7 days)	540	cTnI	Prior to induction of anaesthesia and at termination of CPB	cTnI level > 0.495 ng/L (> 9.9× URL for assay) measured at the end of CPB was predictive of in-hospital adverse outcomes (MI/death)
Lasocki *et al.*^45^	Single centre prospective study; CABG or valve surgery (Acute MI < 7 days were excluded)	502	cTnI ECG changes	20 h post-op	cTnI < 32.5× URL ∼2.5% in hospital mortality cTnI ≥ 32.5× URL ∼22.5% in hospital mortality cTnI > 100× URL 44% in hospital mortality
Thielmann *et al.*^46^	Single centre prospective study: CABG only	2,078	cTnI	1, 6, 12,24 h post op	cTnI was a more sensitive and specific marker of graft failure at a level above 21.5 ng/mL (> 43× URL ng/mL) at 12 h and 33.4 ng/mL (>66.8× URL) at 24 h, compared to myoglobin and CK/CK-MB. CK-MB and EKG changes (ST-segment deviations or new Q wave) did not predict graft failure
Paparella *et al.*^47^	Prospective Single centre study; CABG only (Patients with UA/MI < 7 days included)	230	cTnI	Pre-op, 1,6,12,24 and 36 h post-op, daily from day 2 to 7	cTnI >260× URL (13 ng/L) predicted in-hospital mortality but not 2 year mortality; Peak cTnI generally observed 24 h after surgery
Onorati *et al.*^9^	Prospective single centre study; CABG only	776	cTnI ECG changes (New Q wave or reduction in R waves > 25%) & ECHO feature of MI	Pre-op and 12, 24, 48 and 72 h post-op	cTnI >3.1 μg/L (> 310× URL) at 12 h predicted increased in-hospital and 12 month mortality; Additional ECG and ECHO criteria of MI predicted worst outcome
Thielmann *et al.*^31^^,^^48^	Prospective single centre study CABG only patients undergoing re-angiography post-op	94	cTnI CK-MB	Pre-op, 1, 6, 12, 24, 36 and 48 h post-op	cTnI was the best discriminator between PMI ′in general′ and ′inherent′ release of cTnI after CABG with a cut-off value of 10.5 ng/mL (> 21× URL) and between graft-related and non-graft-related PMI with a cut-off value of 35.5 ng/mL (>71× URL). CK-MB level and ECG changes/TEE could not differentiate between those with or without graft failure.
Croal *et al.*^49^	Prospective CABG+ valve/other cardiac surgery	1365	cTnI ECG changes	2 and 24 h	cTnI at 24 h best predictor ≥53× URL 2.37 OR 30-day mortality, 2.94 OR 1 yr mortality, 1.94 OR 3 yr mortality ≥27× URL 1.05 OR 30-day mortality, 1.14 OR 1 yr mortality, 1.37 OR 3 yr mortality
Provenchère *et al.*^50^	Prospective single centre study CABG and/or valve surgery	92	cTnI	20 h post op	cTnI levels were not predictive of 1 year mortality in a multivariate model.
Fellahi *et al.*^51^	Prospective single centre study; CABG only	202	cTnI	Per-op and 24 h post-op	cTnI ≥ 13 ng/mL (≥ 21.66 x URL) did not predict in-hospital mortality, but was predictive of 2 year mortality (18% vs. 3%; OR 7.3). Best cut off to predict death ranged from 12.1 to 13.4 ng/mL (20.16–21.66× URL)
Adabag *et al.*^34^	Retrospective analysis CABG and/or valve surgery	1186	cTnI CK-MB	Ever 8 h for 24 h post-op, longer if no peak in 24 h	cTnI level independently associated with operative (30 day) mortality; CK-MB had a weaker association with operative mortality
Muehlschlegel *et al.*^26^	Prospective single centre study CABG only surgery	1013	cTnI	Daily from day 1 to 5	24 h cTnI rise ≥ 138× URL HR 2.8 for 5 yr mortality cTnT at 24 h were independent predictors of 5 year mortality in a multivariate model (No additional benefit of measuring cTn beyond 24 h). ECG changes alone did not predict 5 year mortality.
Petaja *et al.*^41^	Meta-analysis CABG and/or Cardiac surgery	2348–3271	cTnI	Up to 7 days post op	Short-term mortality (<6 mths) 8.1% ≥ 21× URL vs. 1.5% <21× URL Long-term mortality (6–36 mths): 10.6% vs. 3.1% (RR 1.06–11.00%)
Hashemzadeh *et al.*^52^	Prospective single centre study CABG +/- Valve surgery (Excluded MI within 7 days)	320	cTnI	Immediately and 20 h post-op	20 h post-op cTnI had better prognostic value than immediate post-op levels. 20 h cTnI level was an independent predictor of in-hospital mortality above a value of 14 ng/mL (>10× URL)
Van Geene *et al.*^53^	Registry retrospective analysis;CABG and/or valve surgery	938 (Separate validation subset, n = 579)	cTnI	1 h post-op	1 h post-op cTn values correlated with hospital mortality with the best cut-off value of 4.25 μ/L (Type of assay and URL for assay not known)
Domanski *et al.*^29^	Meta-analysis CABG only	18,908	cTnI	<24 h post op	5 to < 10× URL 1.00 RR of 30 d mortality 10 to < 20× URL 1.89 RR of 30 d mortality 20 to < 40× URL 2.22 RR of 30 d mortality 40 to < 100× URL 3.61 RR of 30 d mortality ≥100× URL 10.91 RR of 30 d mortality
Ranasinghe *et al.*^27^	Retrospective analysis of 2 prospective randomized controlled clinical trials	440	cTnI	6, 12, 24, 48, 72 h post-op	cTnI levels at 12, 24, 48 and 72 h were all independent predictors of mortality HR ranging from 1.02 to 1.10 for these time points (>4.8 yr follow-up period). Cumulative area under to curve for cTn release up to 72 h was the best predictor of mortality in this model (HR 1.45). Peak cTnI of > 13 ng/mL (URL not defined) did not predict mid-term mortality.

AUC, area under the curve; CABG, coronary artery bypass grafting; CMR, cardiac MRI; CK-MB, creatine kinase-MB fraction; d, day; ECG, electrocardiogram; ECHO, echocardiocardiogram; HR, hazards ratio; h, hour; LGE, late gadolinium enhancement; LV, left ventricle; MACE, major adverse cardiac events; MI, myocardial infarction; mth, month; ng, nanogram; ONBEAT, on-pump beating heart; CABG ONSTOP, on-pump CABG; OR, odds ratio; post-op, post-operative; PMI, perioperative myocardial injury; RR, relative risk; TEE, transoesophageal echocardiogram; cTnI, Troponin I; cTnT, Troponin T; UA, unstable angina; URL, upper reference limit; yr, year.

In summary, we recommend, that for patients with a pre-operative cTn <1× URL, isolated elevations of ‘standard’ cTn assays (cTnT ≥7× URL and cTnI ≥20× URL) within the 48 h post-operative period (in the absence of ECG/angiographic or other imaging evidence of MI), may be indicative of prognostically significant PMI, and require further clinical evaluation to determine whether there is evidence for Type 5 MI. This is particularly so if there is additional clinical evidence for MI such as disproportionate chest pain, unusual ECG changes or new regional wall motion abnormalities on echocardiography in a territory that is dependent on a graft, or dependent on a major ungrafted vessel. However, these threshold values for cTnT and cTnI in defining prognostically significant PMI, may vary from site to site and the actual cTn assay used, and should be established for individual sites. Also, it is important to note that isolated elevations in cTn below these thresholds may still be clinically significant, but their impact on post-CABG mortality appears to be small. For patients with additional ECG/angiography/imaging evidence of MI, an elevation of cTnT or cTnI ≥10× URL should be used to define Type 5 MI, as per the 3rd Universal Definition of MI. For the newest generation of high-sensitive cTn assays, the threshold level above which clinical outcomes post-surgery can be predicted remains to be determined.

### Other biomarkers for quantifying peri-operative myocardial injury

As mentioned above, cTn elevations between 24 and 48 h have been most clearly shown to correlate with mortality post-CABG surgery. However, this may be too late to identify prognostically significant PMI or Type 5 MI, as interventions at this stage may fail to salvage a substantial volume of myocardium at risk. Also, cTn elevation in this early time period (<24 h) may be due to non-ischaemic causes, making it a less reliable marker of regional ischaemia in the first 24 h.

Newer cardiac biomarkers are therefore needed to improve the diagnosis of PMI following CABG surgery with respect to earlier diagnosis, and improving specificity for regional ischaemia, thereby allowing prompt implementation of medical or surgical treatment and to maximise myocardial salvage. Myoglobin, heart-type fatty acid–binding protein,^55^^,^^56^ copeptin,^57^ microRNAs (miR-499 and miR-1),^58^^,^^59^ and cardiac myosin-binding protein C^60^ have been shown to be associated with PMI following CABG surgery. Some of these are not specific for myocardial necrosis, but they seem to provide additional power in combination with conventional cardiac biomarkers for detecting PMI following CABG surgery. Interestingly, new peptides have been identified via a phage display peptide library screen that might be useful in the future to predict PMI after CABG surgery.^49^ Although these new biomarkers seem to be extremely sensitive for detecting PMI, technological improvements for early detection, and large validation cohorts are needed to speed-up their clinical application.

## Role of electrocardiogram for detecting type 5 myocardial infarction following coronary artery bypass graft surgery

The appearance of new Q waves or LBBB on ECG following CABG surgery remain part of the diagnostic criteria for Type 5 MI.^5^ Using ECG, the incidence of Type 5 MI is in the range of 5 to 14%. New ST-segment elevation or depression may indicate ongoing regional ischaemia, and warrant further diagnostic work-up. However, in many post-surgical patients the ECG may not be interpretable, and ECG changes may be non-specific or transient. A number of clinical studies have found that ECG changes alone are not always predictive of poorer outcomes following CABG surgery,^23^^,^^26^^,^^49^ although the additional presence of ECG evidence of PMI with an elevation in cTn appears to be associated with significantly worse outcomes.^8^^,^^9^ Interestingly, a number of studies have shown that many cases of Type 5 MI detected by CMR occur in the absence of new ECG changes (Q waves or LBBB), illustrating the difficulties in relying on ECG changes to detect Type 5 MI.^15^^,^^61^

## Role of cardiac imaging for detecting type 5 MI following coronary artery bypass graft surgery

Although several cardiac imaging modalities exist for detecting new loss of viable myocardium or new regional wall motion abnormalities following CABG surgery, only coronary angiography allows for immediate final decision making (conservative, vs. redo CABG vs. percutaneous coronary intervention).

### Echocardiography to detect type 5 myocardial infarction following coronary artery bypass graft surgery

Echocardiography is the most practical imaging modality for detecting new RWMA following surgery.^13^ However, image quality can be reduced after CABG surgery, due to the presence of pleural or pericardial effusions, inflammation or assisted ventilation, and in these cases transoesophageal echocardiography may be preferable.^62^ Endocardial visualisation might also be enhanced by the use of contrast agents, especially when 2 or more myocardial segments are not visualised by standard echocardiography.^63^ Moreover, detection of RWMA might be improved by more advanced echocardiography imaging modalities such as tissue Doppler imaging or speckle tracking.^64^ However, a large retrospective analysis found that RWMA detected by TEE were not able to predict those patients with graft failure as documented by coronary angiography.^65^ One major limitation of echocardiography is that new RWMA may reflect conditions not necessarily associated with Type 5 MI and include acute ischaemia (without infarction), stunning or hibernation, and non-ischaemic conditions, such as inflammation.

### Myocardial nuclear imaging and cardiac computed tomography to detect type 5 myocardial infarction following coronary artery bypass graft surgery

Radionuclide single-photon emission computed tomography (SPECT) and positron emission tomography (PET) imaging can allow the direct assessment and quantification of myocardial viability before and after CABG surgery,^66^^,^^67^ although given the relatively low spatial resolution of this imaging technique, small areas of non-viable myocardium (especially subendocardial MI), which are commonly found with Type 5 MI, may be missed. Other radionuclide imaging approaches are currently under intense investigation, and will likely be tested in the next few years.^68^

New loss of viable myocardium may be also visualised by cardiac CT.^69^ Multi-slice CT coronary angiography is another useful non-invasive imaging modality that can be utilized to evaluate graft patency following CABG surgery.^10^^,^^11^^,^^70^^,^^71^ However, the radiation dose and the risks of cumulative ionising radiation need to be weighed against the obvious advantages of an early and accurate diagnosis.^72^

### Cardiac magnetic resonance to detect type 5 myocardial infarction following coronary artery bypass graft surgery

Cardiovascular magnetic resonance (CMR) imaging is a well validated imaging technique with high spatial resolution, for the accurate assessment of both myocardial function and viability, which has proven to be an excellent tool in the diagnosis of Type 5 MI.^73^ The presence of new areas of late gadolinium enhancement (LGE), on CMR performed in the first couple of weeks following CABG surgery can detect the presence of new non-viable myocardial tissue required for diagnosing Type 5 MI (see *Table 6*). These clinical studies suggest that Type 5 MI occurs in 20–30% of all patients undergoing elective CABG surgery. Interestingly, the pattern of LGE observed on CMR post-CABG surgery reflects the multi-factorial aetiology of Type 5 MI with examples of transmural infarction (suggesting native artery or graft failure), subendocardial infarction (suggesting inadequate cardioprotection), and patchy areas of infarction (suggesting coronary microembolisation or non-ischaemic myocardial necrosis).^16^^,^^17^^,^^77^

**Table 6:** Major studies using cardiac magnetic resonance to assess Type 5 myocardial infarction following coronary artery bypass graft surgery

Study	Number of patients	Type of surgery	Cardiac biomarkers	Incidence of MI (LGE on CMR)	Major findings
Steuer *et al.*^17^	23	CABG	CKMB/cTnT/cTnI Days 1, 2, and 4 after surgery	18/23 (78%) CMR 4–9 days	First study to use CMR to visualise PMI following CABG surgery. Median LGE mass in patients with PMI was 4.4 g (2.5% of LV). Mixed pattern of LGE with transmural, subendocardial and patchy features. Moderate correlation between elevations in CK-MB, cTnT, cTnI at day 1 and LGE mass. Four patients with transmural LGE all had CK-MB ≥5× URL No pre-op CMR scan performed which may explain the higher than expected incidence of LGE on post-surgery CMR.
Selvanayagam *et al.*^15^	53	CABG (on pump vs. off pump)	cTnI At 1, 6, 12, 24, 48 and 120 h after surgery	9/26 (35%) (on pump) CMR day 6 (range 4–17) 12/27 (44%) (off pump) CMR day 6 (range 4–17)	New median LGE mass in patients with PMI was 6.3±3.6 g on pump and 6.4 ± 4.0 g off pump Moderate correlation between elevations in AUC cTnI and LGE mass (*r*^2^ = 0.4). Only 4 of the 21 patients with LGE on CMR had new Q waves on ECG. Pre-op CMR revealed 47–53% patients had LGE prior to surgery (mean LGE mass 19 g).
Pegg *et al.*^16^^,^^74^	40	CABG (ONBEAT—on pump beating heart vs. ONSTOP—on pump cardioplegia)	cTnI and CK-MB At 1, 6, 12, 24, 48, and 120 h after surgery	6/17 (35%) (ONBEAT) CMR day 6 or 7 (range 6–11.5) 12/23 (52%) (ONSTOP) CMR day 6 or 7 (range 6–11.5)	New median LGE mass in patients with PMI was 8.2 ± 5.2 g ONSTOP and 9.8 ± 9.0 g ONBEAT Good correlation between AUC and 24 h cTnI, CK-MB and new LGE mass. Mixed pattern of LGE with transmural and subendocardial features. Pre-op CMR revealed 100% patients had LGE prior to surgery. cTnI value >6.6 µg/L (165× URL) at 24 h detection of Type 5 MI on LGE-CMR. cTnI better than CK-MB for quantifying myocardial injury
Lim *et al.*^61^	28	CABG	cTnI and CK-MB At 1, 6, 12, 24 h after surgery	9/28 (32%) CMR day 7 (4–10)	cTnI > 83.3× URL at 1 h and peak cTnI/CK-MB at 24 h correlated with new LGEcTnI better than CK-MB in predicting new LGE at both 1 and 24 hNone of the 9 patients with new LGE had Q waves on ECGPre-op CMR performed
van Gaal *et al.*^75^	32	CABG	cTnI and CK-MB At 1, 6, 12, 24 h after surgery	9/32 (28%) CMR day 7 (4–10) and 6 months.	New mean LGE mass 8.7 g on acute scan—no significant change in LGE mass at 6 months There was a strong correlation between the absolute peak cTnI 24 h post-procedure and LGE. Pre-op CMR performed
Alam *et al.*^76^	69	CABG (Elafin vs. placebo)	cTnI At 2, 6, 24 and 48 h after surgery	25% CMR day 5	No difference in AUC cTnI or new LGE mass with Elafin (potent endogenous neutrophil elastase inhibitor—an anti-inflammatory agent) No data on LGE mass given Pre-op CMR performed
Hueb *et al.*^14^	136	CABG (on pump vs. off pump)	cTnI and CK-MB At 6, 12, 24, 36, and 48 h after surgery	13/69 (19%) (on pump) CMR day 6 14/67 (21%) (off pump) on CMR day 6	No data on LGE mass given CK-MB better than cTnI in predicting patients with LGE following CABG surgery The best cut-off for cTnI in predicting Type 5 MI (new LGE on CMR) for on-pump CABG was 162.5× URL and for off-pump CABG was 112.5× URL. The best cut-off for CK-MB in predicting LGE (Type 5 MI) for on-pump CABG was 8.5× URL and for off-pump CABG was 5.1× URL. New Q waves in ECG present in only 7/136 (5%) patients Pre-op CMR performed

AUC, area under the curve; CABG coronary artery bypass grafting; CMR, cardiac MRI; CK-MB, creatine kinase-MB fraction; d, day; ECG, electrocardiogram; ECHO, echocardiocardiogram; HR, hazards ratio; h, hour; LGE, late gadolinium enhancement; LV, left ventricle; MACE, major adverse cardiac events; MI, myocardial infarction; mth, month; ng, nanogram; ONBEAT, on-pump beating heart; CABG ONSTOP, on-pump CABG; OR, odds ratio; post-op, post-operative; PMI, perioperative myocardial injury; RR, relative risk; TEE, transoesophageal echocardiogram; cTnI, Troponin I; cTnT, Troponin T; UA, unstable angina; URL, upper reference limit; yr, year.

Overall, there is a good correlation between elevations in cardiac biomarkers post-surgery and new LGE mass quantified by CMR (see *Table 6*). However, in some patients with absence of LGE on CMR, there was still a significant elevation in AUC cTnI, suggesting that not all post-operative cTnI release represents irreversible myocardial injury,^15^ or that the tissue loss was too small to be detected by CMR.^78^ Therefore, the prognostic significance of post-surgical elevations in cardiac biomarkers in the absence of MI on LGE-CMR remains to be determined. One study has demonstrated that a single cTnI value at 1 h post-surgery accurately predicted new LGE on CMR, increasing the clinical utility of measuring cardiac biomarkers and implementing a change in management to avoid future complications.^61^

In most patients with LGE on CMR, in-hospital patient management was not changed. In one study, a rise in both CK-MB and cTnI to >5× URL in patients with new LGE on CMR had an inverse linear relation with lack of improvement in global left LV function post-CABG surgery, and a pooled analysis of percutaneous coronary intervention (PCI) and CABG patients suggested that new LGE on CMR increased by three-fold the risk of MACE- death, non-fatal MI, admission to hospital for unstable angina or worsening heart failure, or occurrence of ventricular arrhythmia (defined as ventricular fibrillation or sustained ventricular tachycardia).^79^ At least one clinical study^76^ has used the mass of LGE on CMR as a surrogate endpoint to assess the cardioprotective efficacy of a novel therapy during CABG surgery, although in this particular study the anti-inflammatory agent, Elafin, failed to reduce the mass of LGE (*Table 6*).

In summary, LGE-CMR post-CABG surgery has provided important insights into the pathophysiology of Type 5 MI. From a clinical perspective however, its utility for diagnosing Type 5 MI is limited given that it is not widely available, and may be impractical in the early post-operative phase.

## Managing the patient with peri-operative myocardial injury and type 5 myocardial infarction

There is limited evidence from clinical studies comparing strategies on how best to manage either prognostically significant PMI or Type 5 MI following CABG surgery. The key issue in the immediate post-operative period is to identify patients with regional ischaemia due to graft-failure or an acute coronary event in the native coronaries, as this group of patients may benefit from urgent revascularisation.^80^ Graft failure post-CABG surgery is associated with higher mortality (∼15%),^81^ and is potentially amenable to intervention (PCI or redo-CABG).^80^ Early intervention in these patients may reduce the extent of Type 5 MI, thereby improving clinical outcomes.^81^ For non-graft-related PMI, there is currently no specific therapy available, only general supportive measures.

### General management of peri-operative myocardial injury and type 5 myocardial infarction

General supportive measures apply both to graft-related as well as non-graft-related PMI and Type 5 MI. It is important to note that while there are several risk-stratification models to determine the risk of mortality in the patients undergoing CABG surgery based on pre-operative risk factors, such as EuroSCORE, EuroSCORE II, and STS score, there are currently no validated prediction models to determine which patients are at high-risk of PMI or Type 5 MI following CABG surgery. If patients at high risk of PMI or Type 5 MI can be identified, customised management pathways comprising more aggressive monitoring, investigations and/or treatment approaches may result in improved clinical outcomes. The ultimate treatment would be urgent coronary revascularisation, either interventional or surgical.^80^

Non-graft-related PMI is most often related to inappropriate myocardial protection, excessive surgical manipulation, inflammation, and air or plaque embolisation.^82^ Treatment of anaemia, pain and tachycardia can increase coronary blood flow and/or decrease myocardial oxygen consumption, thereby limiting Type 2 MI. Observational studies have shown an association between transfusion and worse outcome, including infections, ischaemic complications, and mortality.^83^^,^^84^ In contrast, a recent multi-centre randomised trial comparing a liberal (haemoglobin, Hb <9 g/dL) vs. a restrictive (Hb <7.5 g/dL) transfusion threshold in CABG surgery patients, showed a lower 30-day mortality in the liberal group, although it was not the primary outcome of the study.^85^ The incidence of PMI was similar in the two groups, but peak values of cardiac biomarkers were not reported. Two recent large multicentre randomised controlled trials showed no benefit of routine intra-operative high dose dexamethasone or methylprednisolone on major adverse events, and its use did not reduce the incidence of Type 5 MI.^86^^,^^87^ Beta-blockers can be used to treat tachycardia, diminish myocardial oxygen consumption and prevent arrhythmias, and are recommended prior to and early after CABG surgery in practice guidelines,^88^ however, hypotension due to systolic dysfunction or PMI may limit their use.

In cases of overt heart failure, pharmacological haemodynamic optimisation and/or mechanical support may be indicated. Due to safety concerns, inotropes are reserved for patients with inadequate peripheral tissue perfusion or hypotension. The β-agonist dobutamine, phosphodiesterase inhibitors like milrinone or enoximone, and the calcium sensitiser levosimendan can all be used to treat postoperative refractory low cardiac output syndrome and decompensated heart failure.

In patients with insufficient coronary perfusion (before surgery or insufficient graft perfusion), the intra-aortic balloon pump (IABP) may provide improvement of haemodynamics while underlying cause(s) of instability can be addressed and is still being used in high risk patients or in patients with difficulties weaning off cardiopulmonary bypass.^89^ A recent meta-analysis showed benefit of a pre-operative intra-aortic balloon pump insertion in patients undergoing CABG surgery on 30-day mortality, and this may be considered in selected unstable high-risk patient preoperatively.^90^ Advanced mechanical support may be indicated in severe cardiac failure, where inotropes, vasopressors and IABP fail to restore adequate output. Extracorporeal Life Support (ECLS or ECMO) may be a bridge to recovery of cardiac function, or bridge to decisions about further long-term mechanical support (LVAD) and future transplantation. Unfortunately, survival in ECLS treated patients is only 20–40%.^91^

### Managing the patient with suspected graft-related failure

The incidence of early graft failure is ∼3%,^92^ and the rate of graft occlusion before discharge varies from 3 to 12% for vein grafts (3 to 4% for radial arteries and 1 to 2.5% for internal mammary arteries^48^). It is often difficult to distinguish graft-related from non-graft-related PMI and Type 5 MI, and surgeons rely on elevations in cardiac biomarkers, unexplained low cardiac output syndrome (LCOS), persistent ischaemic ECG changes, recurrent ventricular tachycardia and fibrillation, and new echocardiographic RWMAs to detect graft failure following CABG surgery. A variety of patient symptoms and objective findings should raise suspicion of regional ischaemia due to early graft failure, and trigger prompt evaluation with an ECG, measurement of cardiac biomarkers, coronary angiography or other appropriate cardiac imaging. These include the presence of typical or atypical chest pain, unexplained shortness of breath, haemodynamic instability as well as difficulty in weaning off cardiopulmonary bypass, refractory arrhythmia or persistent circulatory failure. Unfortunately, all of the above can be present following CABG surgery, even in the absence of regional ischaemia, hence none of these findings are sensitive or specific enough in isolation to accurately identify the presence of regional ischaemia, and so the appropriate diagnostic or management pathway should be determined in each patient taking the whole clinical picture in consideration. Equally, regional ischaemia may be present even in the absence of the above findings. The assessment of regional ischaemia following CABG surgery remains a considerable challenge for managing PMI and Type 5 MI.

The main cause of early graft failure post CABG surgery is graft occlusion but other causes include graft kinking and anastomotic stenosis.^46^ A graft-related cause is identified in 60–80% of coronary angiograms performed for this indication, and consecutive re-revascularisation is performed in 50–70% of graft-related Type 5 MI.^81^^,^^92–95^ However, in one study, 24–35% of patients undergoing coronary angiography after CABG for early graft dysfunction had patent grafts.^93^ One retrospective series found that an urgent post-CABG coronary angiogram was required in 1.8% patients, and more than half of these patients needed re-intervention, and, in spite of this, had high mortality.^96^ In multi-variate analysis, younger patients, female patients, smaller patients, and patients receiving a combined arterial and venous revascularisation were at a higher risk for an unplanned post-surgical coronary angiogram.^96^

When detected, potentially correctable abnormalities included early graft thrombosis, anastomotic stenosis, bypass kinks, overstretching or tension, significant spasm or incomplete revascularization. Compared with native coronary PCI, bypass graft PCI has been shown to be independently associated with higher in-hospital mortality.^97^ In the CathPCI registry, patients undergoing bypass graft PCI more frequently required intra-aortic balloon pump counter pulsation, longer fluoroscopy time, and larger amount of contrast medium; and less frequently achieved TIMI flow grade 3 post-stenting, were more likely to receive blood transfusions, and had higher rates of post-procedural complications and in-hospital mortality.^97^ In one of the few studies that investigated the appropriate treatment for patients with early graft failure following CABG surgery, the major findings were that: (i) patients with prompt re-intervention for early graft failure after CABG surgery had a higher number of graft/patient failure than in patients managed conservatively; (ii) even with more graft failure per patient, there was a trend towards smaller size of MI in the early aggressive re-intervention group than in the conservative group; and (iii) coronary angiography was a good tool to discriminate the aetiology of postoperative infarction (graft-related or non-graft-related).^81^

Early graft failure has been shown to be associated with a higher elevations in cTnI (about >45× URL at 12 h and >70× URL elevation at 24 h for cTnI).^35^^,^^46^^,^^48^ However, it is important to appreciate that there may be a significant overlap between patients with or without graft failure even at this level of biomarker elevation.^35^^,^^46^^,^^48^ Another important finding from these studies is that ECG and/or imaging evidence of MI did not appear to reliably identify those with early graft failure following surgery. Therefore, high cTnI elevations in the post-surgical period (>45× URL at 12 h and >70× URL elevation at 24 h), even in the absence of ECG and/or imaging evidence of MI, should raise the suspicion of early graft failure. However, it is important to have earlier markers of graft failure to allow the implementation of a change in management in order to limit PMI and improve clinical outcomes post-CABG surgery. In this regard, some studies have shown that post-operative cTn levels at 1 h post-surgery may be used to predict Type 5 MI on CMR, but the role of this measurement in detecting early graft failure has not been investigated.^61^ The detection of graft dysfunction by intraoperative transit time flow measurement (TTFM) within the graft may allow early detection of graft failure and thereby provide a potential strategy for limiting PMI and Type 5 MI.^98^^,^^99^ In addition, this approach has been shown to predict graft failure at 1 month^100^ and 6 months post-CABG surgery.^101^

In summary, strategies aimed at earlier identification of patients with significant on-going regional ischaemia could salvage viable myocardium. Anaesthesiologists and intensivists should be involved in this process. Early coronary angiography and on-site consultation of an interventional cardiologist and cardiac surgeon should result in a decision on the management of the individual patient, taking into account the extent of ischaemia, coronary anatomy, and comorbidities.

**Figure 3:**
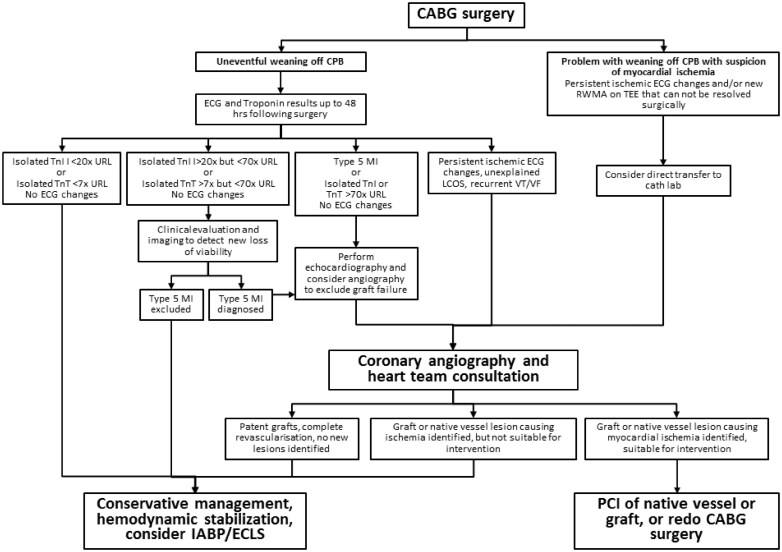
Proposed algorithm for managing patients with possible peri-operative myocardial injury and Type 5 myocardial infarction following coronary artery bypass graft surgery. CPB, cardiopulmonary bypass; RWMA, regional wall motion abnormality; TEE, transeophageal echocardiography; LCOS, low-cardiac output syndrome; VT, ventricular tachycardia; VF, ventricular fibrillation; IABP, intra-aortic balloon pulsation; ECLS, Extracorporeal Life Support; URL, upper reference limit.

We present a management algorithm (*Figure 3*) providing guidance on when to perform coronary angiography for suspected PMI or Type 5 MI. It proposes emergent coronary angiography in case of clear signs of acute myocardial ischaemia or unexplained haemodynamic compromise immediately post-surgery, and urgent coronary angiography in case of recurrent ventricular arrhythmias, unexplained LCOS or persistent ischaemic ECG changes. Furthermore, high cTn elevations in the post-surgical period (such as cTnI >45× URL at 12 h and >70× URL elevation at 24 h) even in the absence of ECG and/or imaging evidence of MI, should raise the suspicion of early graft failure. This proposed algorithm aligns well with the current ESC/EACTS guidelines on myocardial revascularization (2014), which support emergency PCI in early post-operative graft failure to limit the extent of myocardial injury.^80^ Additionally, the current ESC/EACTS guidelines favour PCI to the body of the native vessel or IMA graft while avoiding PCI to an occluded vein graft or graft anastomosis site and reserve re-do surgery to patients with coronary anatomy unsuitable for PCI.^80^ Future studies aiming at earlier and more precise identification of patients with suspected graft-related ischaemia should allow one to refine this algorithm further.

### Decision making following coronary angiography post-surgery

Once coronary angiography following CABG in cases of suspected graft failure, the treatment strategy (conservative vs. revascularisation) depends on many factors, and the decision needs to be made in close consultation with the Heart Team (intensivists, surgeons and cardiologists). These factors include the coronary anatomy, graft occlusion vs. native vessel occlusion, extent of myocardial ischaemia, extent of viable myocardium, clinical symptoms, haemodynamic status and inotrope support, and age and co-morbidities.

A conservative strategy should be considered if:

- All grafts are patent.
- There are no lesions in native coronary arteries potentially involved in post-operative myocardial ischaemia.
- The graft or native coronary artery occlusion was identified late, in which case consider viability assessment first.
- In cases of venous graft occlusion anastomosed on non-major left anterior descending (LAD) coronary artery with no lesion suitable for PCI on the related native coronary artery.

Revascularisation by PCI should be considered if:

- There is early graft dysfunction.
- There are suitable lesions in native coronary arteries involved in the post-operative myocardial ischaemia.
- In the presence of severe cardiogenic shock emergency PCI or ECLS should be considered.

If PCI is chosen there are certain risks and technical challenges. PCI should be performed on lesions in the native vessels supplying the ischaemic region, and should be avoided in the occluded vein graft or graft anastomosis site, except when lesions on the native vessels are not suitable for PCI.

Revascularization by redo CABG surgery should be considered if:

- The coronary anatomy is unsuitable for PCI
- There is involvement of a large extent of ischaemia (e.g. LAD territory).
- There is failure of LIMA or a Y-graft to the left system.

If redo CABG is being considered there are certain risk and technical challenges. Recurring cardiopulmonary bypass (CPB) with cardioplegic arrest may intensify acute myocardial ischaemia-reperfusion injury, already sustained, and a period of recovery using ECLS, may be beneficial in the initial 24–48 h after treatment. Redo CABG surgery may also be considered using ‘beating heart surgery’ (without cardiac arrest and cardioplegia) under cardiopulmonary bypass support, in order to limit additional acute myocardial ischaemia-reperfusion injury.

## Using peri-operative myocardial injury and type 5 myocardial infarction to assess the cardioprotective efficacy of novel therapies in the setting of coronary artery bypass graft surgery

Cardioprotective strategies such as ischaemic preconditioning (IPC), ischaemic post-conditioning (IPost), remote ischaemic preconditioning (RIPC), and a number of drugs including volatile anesthetics which recruit the signal transduction pathways underlying conditioning, have been shown to attenuate myocardial injury following acute ischaemia-reperfusion injury.^102–108^ Ischaemic cardioplegic arrest on cardiopulmonary bypass with subsequent reperfusion was therefore considered an ideal and well controlled clinical setting to translate findings from animal experiments to humans. In fact, a number of smaller studies have reported reduced MI size with IPC, IPost, and RIPC (for review see reference 102), and cyclosporine A.^109^^,^^110^ These studies used biomarker release (CK, CK-MB, and cTn) to quantify PMI. It is important to note that the majority of studies have measured the magnitude of PMI to assess the cardioprotective efficacy of novel therapies, and did not investigate whether the new intervention was able to reduce the incidence of Type 5 MI or mortality. Two moderately sized trials also reported improved clinical outcomes with RIPC at short-^111^ or more long-term^112^ as a secondary endpoints.

In contrast to these encouraging phase II a studies, two recent larger phase III trials assessing RIPC neither confirmed reduced biomarker (cTnT or cTnI) release nor improved clinical outcomes during hospitalization^113^ or at one year follow-up.^114^ In both these neutral trials, less than 50% of patients had only CABG surgery, and the others had either additional or only valvular surgery. Valvular surgery causes greater traumatic injury than CABG, and the contribution of trauma to total biomarker release may have diluted a potential cardioprotective effect of remote ischaemic preconditioning. In contrast to these larger trials, the original positive phase II trials had only recruited patients undergoing CABG surgery.^112^^,^^115^ There are also other causes of biomarker release such as bypass graft failure^48^ or microembolization of atherothrombotic debris,^77^ which are not associated with subsequent reperfusion injury and from which, therefore, no protection by conditioning or drugs is expected. More disconcerting than the lack of reduction in biomarker release is the lack of improved clinical outcomes, which retrospectively also confirms the lack of reduced biomarker release in the two recent phase III trials.^116^ Therefore, the search for novel biomarkers specific to cardioprotection by ischaemic conditioning such as protectomiRs^117^ is of particular interest.

## Recommendations for defining and managing prognostically significant peri-operative myocardial injury

In this ESC Joint WGs Position paper, we have provided recommendations for defining prognostically significant PMI (*Table 7*). In summary, we would recommend that isolated elevations in cTnT ≥7× URL and/or cTnI ≥20× URL in the 48-h post-operative period may indicate the presence of prognostically significant PMI, and should prompt clinical evaluation to exclude Type 5 MI. Where ECG/angiography/imaging evidence of MI is available, lower levels of biomarker elevation (cTn x10 URL) should be considered for diagnosing prognostically significant PMI, as per the Universal MI definition.

**Table 7:** Overview of definitions for peri-operative myocardial injury and Type 5 myocardial infarction

Diagnostic criteria	Cardiac biomarker	Threshold for isolated elevation in cardiac biomarker (with no ECG or imaging changes of MI)	Threshold for elevation in cardiac biomarker with ECG and imaging changes of MI
Universal definition^13^ Type 5 MI	Troponins only	N/A	≥10× URL
Universal definition^5^ Peri-operative myocardial injury	Troponins only	<10× URL	N/A
SCAI^18^ Clinically relevant MI	CK-MB and Troponins	≥10× URL (CK-MB) ≥70× URL (cTn)	≥5× URL (CK-MB) ≥35× URL (troponin)
ESC Joint WG Criteria Prognostically significant peri-operative myocardial injury	Troponins only	≥7× URL (cTnT) ≥20× URL (cTnI) (Does not apply to hs-cTnT)	≥10× URL

URL, upper reference limit.

We have also proposed an algorithm for managing CABG patients with or without suspected graft failure based on elevations in cardiac biomarkers (*Figure 3*). Isolated elevations in cTn (>70× URL in the 48 h post-operative period), even in the absence of any other feature of MI, may be indicative of graft failure and warrant further investigation with coronary angiography and re-revascularization by PCI or CABG surgery if indicated. More studies are needed to establish thresholds, especially for hs-cTnT elevations, which can be used in conjunction with clinical features and imaging findings, to predict those patients with regional ischaemia or graft failure. Furthermore, studies are required to better define the role of coronary angiography post-CABG surgery to detect early graft failure.

## Funding

European Cooperation in Science and Technology (COST EU-ROS) and Hungarian Scientiﬁc Research Fund (OTKA K 109737 and ANN 107803) to P.F; British Heart Foundation (grant number FS/10/039/28270), the Rosetrees Trust, and National Institute for Health Research University College London Hospitals Biomedical Research Centre to D.J.H.; Italian Ministry of Health (GR-2009-1596220) and the Italian Ministry of University (RBFR124FEN) to C.P.; Netherlands Organization for Health Research and Development (ZonMW Veni 91612147) and Netherlands Heart Foundation (Dekker 2013T056) to L.V.L.; German Research Foundation (He 1320/18-3; SFB 1116 B8 to G.H.).

**Conflict of interest**: D.H., M.T., V.S., J.B., G.K., R.M., J.S., F.P., P.K., P.M., N.A., S.L., C.P., G.B., J.O., U.F., M.C., U.F., J.F.O., C.M., L.V.L., M.S.N. have no disclosures. G.H. served as consultant for Servier. P.F. is an owner of Pharmahungary Group, a group of R&D companies.
